# Ileosigmoid knotting: A review of 923 cases

**DOI:** 10.12669/pjms.38.3.5320

**Published:** 2022

**Authors:** Sabri Selcuk Atamanalp, Esra Disci, Rifat Peksoz, Refik Selim Atamanalp, Cansu Tatar Atamanalp

**Affiliations:** 1 Prof. Sabri Selcuk Atamanalp, MD. Department of General Surgery, Faculty of Medicine, Ataturk University, Erzurum, Turkey; 2 Esra Disci, MD. Assistant Professor, Department of General Surgery, Faculty of Medicine, Ataturk University, Erzurum, Turkey; 3 Rifat Peksoz, MD. Assistant Professor, Department of General Surgery, Faculty of Medicine, Ataturk University, Erzurum, Turkey; 4 Refik Selim Atamanalp, MD. Assistant, Department of Medical Pathology, Prof. Dr. Cemil Tascioglu City Hospital, Istanbul, Turkey; 5 Cansu Tatar Atamanalp, MD. Assistant, Department of Pediatrics, Haseki Education and Research Hospital, Istanbul, Turkey

**Keywords:** Ileum, Sigmoid colon, Ileosigmoid knotting, Intestinal obstruction

## Abstract

**Objectives:**

Ileosigmoid knotting (ISK) is a rare intestinal obstruction form worldwide. The aim of this study was to investigate changing trends in ISK.

**Methods:**

The Web of Science and PubMed databases were electronically searched to find all publications to evaluate all epidemiological, etiological, clinical, laboratory, radiological, therapeutic, and prognostic factors in ISK.

**Results:**

Most of the cases were reported from Asian and African countries. Mean age was 43.9 years with a 79.9%/20.1% of male/female ratio. Main symptom period was 48.1 hours, while the most common clinical features were abdominal pain/tenderness (99.1%), distention (88.3%), and obstipation/constipation (58.8%). Abdominal X-ray radiography, computerized tomography (CT), and magnetic resonance imaging (MRI) were diagnostic in 8.2%, 96.2%, and 100.0%, respectively, while the total diagnostic accuracy rate was 20.8%. Bowels were gangrenous in 85.6% of the patients. Ileum resection was applied in 14.0% of the cases, while sigmoid colon resection in 7.6%, and both segment resection in 67.1%. The mortality rate was 22.7%, while the morbidity rate was also 22.7%.

**Conclusion:**

ISK is a rare disease, but it is still catastrophic despite its two-century recognised past. As an exception, diagnostic convenience arising from CT or MRI looks like the most important change over the last half-century.

## INTRODUCTION

Ileosigmoid knotting (ISK) is the wrapping of the terminal ileum or sigmoid colon around the other structure causing a double-loop intestinal obstruction. Although ISK is a rare clinical entity worldwide, its incidence is relatively high in Asia, Africa, Middle East, South America, and Eastern and Northern Europa.^1^ The incidence of ISK is also relatively high in Eastern Anatolia, my practicing area.^2^ My colleagues and I have 80 cases of experience with ISK, over 55 years from June 1966 to July 2021. This is one of the largest single-center ISK series in totally a few hundred cases reported to date in the literature documented in the Web of Science^3^ and PubMed^4^ databases. In light of our comprehensive experience, we wanted to evaluate all achievable ISK cases documented worldwide to investigate changing trends from past to present.

## METHODS

The Web of Science and PubMed databases were electronically searched to find publications related to ISK. Subsequently, the papers were utilized according to year, country, case number, age, gender, symptoms, signs, laboratory tests, radiological studies (X-ray, computerized tomography-CT, and magnetic resonance imaging-MRI), diagnostic accuracy, treatment, predisposing factors, and prognosis. Publications, of which full texts or summaries were not obtained, were excluded from the study. Similarly, correspondences, letters, and repetitive case reports or case series, which didn’t present a new case or cases, were not included.

This study was approved by the institutional review board (Ethical Committee of Ataturk University Faculty of Medicine, B.30.2.ATA.0.01.00/219).

## RESULTS

Although at least 204 cases have been reported from England, Finland, Russia, Sri Lanka, India, Uganda, South Africa, Rhodesia, Ghana, and United States from the first description of ISK by Rokitansky in 1836 to 1967,^5^,^6^ unfortunately, some details of these reports excluding case numbers or countries, were not found in the Web of Science^3^ or PubMed^4^ databases. According to available data, from the first publication by Shepherd^5^ in 1967 to date, 114 manuscripts were published on ISK, of which 82 were uploaded to the Web of Science,^3^ while 102 to the PubMed.^4^ In this paper, a total of 719 cases were considered in detail in 73 papers providing inclusion criteria, 21 of which were case series (≥5 cases), while there were eight small case series (2-4 cases) and 44 case reports (one case).^1^,^2^,^5^-^25^

The current total ISK case number aggregated to 923 all over the world by this time. The state of origin was achieved in 839 patients. According to data, the maximum patient number was reported from Turkey (213 patients, 25.4%), while the following countries were noted as Uganda 118, Malawi 99, Finland 73, Kenya 61, Ethiopia 60, India 44, Tanzania 24, Zimbabwe 23, South Africa and Ivory Coast 16, Canada 15, Nigeria 12, Iran 11, England and Morocco eight, Japan six, United States five, Russia and Pakistan four, France and Ghana three, Spain and Rhodesia two, Australia, Scotland, Korea, Oman, Greece, Holland, Botswana, Sri Lanka and Kuwait one.

The detailed data of ISK cases are presented in Table-I. Among evaluated 668 patients, the mean age was 43.9 years (range: two weeks-92 years). The male/female ratio was 532/134 (79.9%/20.1%). The mean symptom period was 48.1 hours (range: 2 hours-16 days) among evaluated 641 patients. In evaluated 580 patients, the most common clinical features were abdominal pain/tenderness in 575 patients (99.1%), distention in 512 (88.3%), obstipation/constipation in 341 (58.8%), and vomiting in 322 (55.5%), while the other findings were rebound tenderness and/or muscular rigidity in 186 (32.1%), hypo/a-kinetic bowel sound in 81 (14.0%), hyperkinetic bowel sound in 34 (5.9%), melanotic stool in 17 (2.9%), and fever in 16 (2.8%). Of these patients, 167 (28.8%) were in shock state.

**Table I:** Preoperative, operative, and postoperative data including the results of 21 case series (≥5 cases), 8 small case series (2-4 cases), and 44 case reports (1 case).

Reference	N	A	M	F	SP	PT	D	OC	V	RM	AS	S	XR	CT	MRI	DA	BG	IE	SE	DE	MT	MB
Shepherd^5^	92	42	78	14	18	92	90	0	0	0	0	0									44	
Vaez-Zadeh and Dutz^6^	11	45.1	10	1	45	11	11	11	0	11	11	11	11	0	0		10	2	0	8	7	2
Young et al^7^	14												14	0	0							
Kakar and Bhatnagar^8^	11	38	10	1		11	3	0	11	0	0	4				2	7	0	0	7	3	0
Puthu et al^9^	7	40.6	3	4	30.7	7	7	0	7	7	0	7	7	0	0	2	7	0	0	7	2	2
N’Gueassan et al^10^	16	42	14	2																		
Gibney and Mock^11^	15	55.6	12	3	48	15	15	15	3	0	0	0	15	0	0	0	7	2	0	5	7	0
Alver at al^12^	68	49	57	11	45.6	68	64	68	59	21	0	38	56	0	0	0	50	10	5	37	21	7
Akgun^13^	16	845	11	5	106	16	15	9	14	11	0	9	16	0	0	0	15	6	3	6	3	1
Kedir et al^14^	9					9	9	9	9	5	0	5					9	0	0	9	4	0
Raveenthiran^15^	7	43.6	6	1	53.1	7	6	7	5		1	4	7	0	0	5	7	0	4	3	0	4
Alver et al^16^	8	39	4	4	62.4	8	8	0	6	0	0	4	8	0	0	0	8	3	0	5	2	3
Kotisso and Bekele^17^	22				83	22	22	22	22	0	0	0					14	0	0	14	3	9
Benerjee et al^18^	9	49.1	8	1	42.7	9	6	9	7	0	0	3	9	0	0	0	9	0	0	9	2	4
Cakir et al^19^	36	55	20	16	55.4	36	36	0	0	0	0		36	0	0	0	36	0	0	36	0	12
Chalya and Mabula^20^	24	48	20	4	144	22	23	16	14	0	0	7	24	0	0	1	23	5	0	18	4	17
Ooko et al^21^	61	35.8	51	10	38.4	60	51	46	48	28	0	10	52	1	0	16	55	9	6	45	7	15
Atamanalp^22^	80	47.5	58	22	43.9	80	77	79	62	38	72	43	58	9	3	11	61	8	10	44	15	16
Purcell et al^23^	99	45.4	87	12	24											52					11	4
Abebe et al^24^	28	41.7	21	7	38	28	24	23	28	19	21	5	17	0	0	6	26	8	1	17	6	11
Mbanje et al^25^	21	37	18	3	27.2	20	0	0	0	21		7	21	0	0	0	21	4	0	17	1	11
Small case series^3^,^4^	21	30.4	10	3	69.5	12	13	7	2	4	1	2	9	2	0	1	14	4	6	8	2	3
Case reports^3^,^4^	44	35.4	34	10	33.4	42	32	20	25	21	9	8	31	14	1	13	37	7	2	30	3	5
Evaluated cases	719	668	666	666	641	580	580	580	580	580	580	580	458	458	458	523	484	484	484	484	647	556
Total or mean		43.9	532	134	48.1	575	512	341	322	186	115	167	391	26	3	109	416	68	37	325	147	126
Range or %		2 w-92	79.9	20.1	2-384	99.1	88.3	58.8	55.5	32.1	19.8	28.8	85.4	5.7	0.7	20.8	86.0	14.0	7.6	67.1	22.7	22.7

**N**: Number, **A**: Age (mean years), **M**: Male, **F**: Female, **SP**: Symptom period (mean hours), **PT**: Pain and/or tenderness, **D**: Distention, **OC:** Obstipation or constipation, **V:** Vomiting, **AS:** Abnormal bowel sound, **RM:** Rebound tenderness and/or muscular rigidity, **S:** Shock, **AR:** X-ray radiography, **CT:** Computrrized tomography, **MRI:** Magnetic resonance imaging, **DA:** Diagnostic accuracy, **BG:** Bowel gangrene, **IE:** Ileum excision, **SE:** Sigmoid excision, **DE:** Double segment excision, **MT:** Mortality, **MB:** Morbidity.

Among evaluated 472 patients, leukocytosis and anemia rates were 15.0% (71 patients) and 2.5% (12 patients), respectively. Radiological investigations were evaluated in 458 patients and abdominal X-ray radiography was obtained in 391 (85.4%) patients with an 8.2% (32/391) of accuracy rate. Computerized tomography (CT) and magnetic resonance imaging (MRI), which were used by 2000s, were obtained in 26 (5.7%) and 3 (0.7%) of the patients, respectively, while these methods were diagnostic in 96.2% (25/26) and 100.0% (3/3) of the patients, respectively. Based on both clinical and radiological findings, the total diagnostic accuracy of ISK was 20.8% (109/523).

In 71 patients (9.9%), some predisposing factors including previous surgery and/or adhesion in 30 patients, gravida in 19 (puerperium two, labor one), internal herniation in 11, mobile cecum and/or rotation anomaly in eight, and Meckel’s diverticulum in three, were determined. ISK was in Type-I (ileum active) in 183 (83.2%) of evaluated 220 patients, while in Type II (sigmoid colon active) in 37 (16.8%). Among evaluated 484 patients, as operative findings, bowels were viable in 68 patients (14.0%), while gangrenous in 416 (86.0%). Gangrene was in the ileum in 64 patients (13.2%), in the sigmoid colon in 28 (5.8%), and in both segments in 324 (66.9%), while gangrene was extended across distal jejunum, cecum or ascending colon in 34 patients (7.0%). As surgical procedures, ileum resection was applied in 68 patients (14.0%), sigmoid colon resection in 37 (7.6%), and both segment resection in 325 (67.1%), while patients with extensive gangrene were additionally needed a wider resection involving gangrenous segments. In 5 patients (1.0%), as a recurrence-reducing procedure, sigmoidopexy was added.

Of evaluated 647 patients, 147 (22.7%) were lost, five of whom during laparotomy. The most common death cause was toxic shock. In 126 (22.7%) of evaluated 556 patients, some complications including wound infection in 53 patients, wound dehiscence in 18, pulmonary problems in 16, anastomosis leakage in 15, hematalogic problems in 13, renal problems in nine, cardiac problems in six, gastrointestinal problems in five, peritonitis in four, stoma problems in three, adhesive ileus in two, and pancreatitis in one, were determined. ISK and sigmoid volvulus recurrence were seen in one patient for each (0.2%), both in patients treated with decompression alone.

## DISCUSSION

According to available data, the present 923-case series comprises the largest multicenter ISK series over the world.^3^,^4^ On the other hand, apart from review articles presenting multicenter data, following a 99-case report by Purcell et al^23^ in 2020 and 92-case report by Shepherd^5^ in 1967, our 80-case series is the third-largest single-center ISK series worldwide.^3^,^4^

Although ISK is rare worldwide, it is relatively common in some Asian, African, Middle Eastern, South American, and Eastern and Northern European countries. This interesting distribution arises from two major anatomical prerequisites: a long ileal mesentery with mobile terminal ileum and an elongated sigmoid colon with a long mesentery, the last which is known as ‘dolichosigmoid’.^1^ These predisposing factors may be congenital, as seen in childhood ISK, or acquired, which arises from dietary habits such as high-fiber diet or overeating following a long starvation.^1^,^5^,^6^,^10^,^12^,^18^,^20^ Otherwise, some conditions including internal herniation, Meckel’s diverticulum, mobile cecum, late pregnancy, adhesions, and intussusceptions may precipitate ISK.^1^,^6^,^16^,^19^,^23^ The disease commonly occurs in men with a peak incidence in the third to fifth decades, while it is rare in childhood.^1^,^6^-^8^,^12^,^19^,^20^

In ISK, the mean symptom period ranges between 2-240 hours with a mean of 24-48 hours.^1^,^6^-^11^,^12^,^14^,^15^-^19^,^24^,^25^ Most likely due to the improvement of healthcare and socioeconomic conditions, this period became shorter in recent years.^3^,^4^,^22^ A severe acute, colicky abdominal pain, distention, and obstipation/constipation, the classical volvulus triad, is observed in 27-100% of patients, while vomiting is less often. Other rare clinical features are melanotic stool and fever. Clinical examination generally reveals abdominal tenderness and abnormal (hyper- hypo- or a-kinetic) bowel sounds. Rebound tenderness and/or muscular rigidity generally suspect bowel gangrene and/or peritonitis.^1^,^6^-^9^,^10^-^12^,^14^,^15^-^20^,^22^,^24^ Apart from a decrease in the symptom period, the clinical presentation of ISK did not demonstrate any change over the last half-century.^3^,^4^,^18^,^22^

Although leukocytosis and acidosis may accompany the clinical appearance in late periods, unfortunately, there is no specific laboratory test to diagnose ISK. Endoscopy may diagnose sigmoid volvulus, but it remains incapable in diagnosing ISK. Abdominal X-ray images generally demonstrate a dilated sigmoid colon on the right side and multiple small intestinal air-fluid levels on the left side.^1^,^6^,^7^,^9^-^13^,^15^-^20^,^22^,^24^,^25^ CT and MRI, the last which is preferred in pregnant women, have high diagnostic values by showing whirl sign in the whirled mesenteries, which is pathognomonic for volvulus.^1^,^21^,^23^,^24^ Following the usage of CT and MRI by 2000s, the diagnostic accuracy rate distinctively increased.^3^,^4^,^22^ Despite the critical condition, the correct diagnosis of ISK is not easy and is less than 20% when CT and MRI are not used.^1^,^23^-^25^ Misdiagnosis generally consist of intestinal obstruction,^9^,^11^-^13^,^15^,^19^-^21^,^22^-^25^ sigmoid volvulus,^9^,^12^,^18^,^23^-^25^ or non-obstructive emergency.^9^,^12^,^15^,^22^-^25^

Due to the volume loss and toxic material absorption arising from intestinal obstruction and bowel ischemia or gangrene, ISK rapidly leads to hypovolemic and/or toxic shock in most patients. For this reason, a rapid and effective resuscitation followed by emergency laparotomy is essential in the treatment of ISK.^2^,^6^,^8^-^10^,^15^-^17^,^22^,^24^,^25^ Although endoscopy demonstrates sigmoid mucosa, it cannot give information about the viability of the ileum. Additionally, the unravelling of the knot is often very difficult or impossible, even sometimes in surgery, which describes ISK as ‘gordian knot’. For this reason, endoscopic decompression is both condemned to fail and contraindicated due to its probable complications, including bowel perforation or missing ileum gangrene.^2^,^11^,^12^ In patients with gangrenous bowel, following the resection of the unviable segments, primary anastomosis is preferred in well-conditioned patients, while stoma is used in bad-conditioned cases.^2^,^6^,^8^-^10^,^12^-^19^,^22^,^24^,^25^ Interestingly, over the last half-century, current surgical developments led to the use of primary anastomosis instead of the stoma in selected patients.^3^,^4^,^6^,^9^,^10^,^12^-^19^,^22^,^25^ If the bowels are viable, decompression alone or in well-conditioned patients, sigmoid colectomy or sigmoidopexy, is advised.^2^,^8^,^11^,^22^

Despite developed surgery and modern pre-, peri- and post-operative care techniques, the prognosis of ISK is still relatively poor, particularly in childhood, elderliness, and pregnancy.^2^,^6^-^10^,^12^,^13^,^15^,^17^-^19^,^22^-^25^ Unfortunately, the prognosis of ISK did not demonstrate any statistical difference over the last half-century.^3^,^4^,^23^-^25^ For this reason, ISK is described as a surgeon’s nightmare.^4^ The mortality rate varies between 0-48%^2^,^9^,^10^,^12^-^15^,^20^,^22^-^25^ The most frequent cause of death is toxic shock.^2^,^9^,^10^,^12^-^14^,^17^,^18^,^20^,^22^,^25^ The development of bowel gangrene worsens the prognosis by increasing this rate from 7-8% to 20-100%,^22^ while the other bad prognostic factors are advanced age and late admission.^2^,^12^,^22^ The morbidity rates are 30-80%^14^,^15^,^20^,^22^,^25^ The most common complications are wound infection or dehiscence, while the others are anastomosis or stoma problems, pulmonary, cardiovascular, renal, gastrointestinal complications, and adhesive ileus.^2^,^6^,^9^,^13^,^15^-^17^,^18^-^20^,^22^-^25^ The recurrence of ISK and sigmoid volvulus is very rare with one case for each reported to date, which develops in just decompressed patients.^11^,^22^

## CONCLUSIONS

ISK is a rare disease, but it is still catastrophic despite its two-century recognised past. As an exception, diagnostic convenience arising from CT or MRI looks like the most important change over the last half-century.

### Authors’ Contribution:

**SSA:** Data collection, manuscript writing.

**ED:** Data collection, revision of the final draft.

**RP:** Data collection, revision of the final draft.

**RSA:** Revision of the final draft.

**CTA:** Revision of the final draft.

**SSA:** Is responsible for responsible and accountable for the accuracy or integrity of the work.
